# From meta-omics to causality: experimental models for human microbiome research

**DOI:** 10.1186/2049-2618-1-14

**Published:** 2013-05-03

**Authors:** Joëlle V Fritz, Mahesh S Desai, Pranjul Shah, Jochen G Schneider, Paul Wilmes

**Affiliations:** 1 Eco-Systems Biology Group, Luxembourg Centre for Systems Biomedicine, University of Luxembourg, Avenue des Hauts-Fourneaux, 7, Esch-sur-Alzette, L-4362, Luxembourg; 2 Translational & Experimental Research Group, Luxembourg Centre for Systems Biomedicine, University of Luxembourg, Avenue des Hauts-Fourneaux, 7, Esch-sur-Alzette, L-4362, Luxembourg; 3 Department of Medicine II, Saarland University Medical Center, Kirrberger Str., Homburg/Saar, D-66421, Germany

**Keywords:** Causality, Diet, Human microbiome, Hypothesis testing, *In vivo* model, *In vitro* model, *Ex vivo* model, *In silico* model, Dysbiosis, Disease, Microfluidics, Host-microbe interactions

## Abstract

Large-scale ‘meta-omic’ projects are greatly advancing our knowledge of the human microbiome and its specific role in governing health and disease states. A myriad of ongoing studies aim at identifying links between microbial community disequilibria (dysbiosis) and human diseases. However, due to the inherent complexity and heterogeneity of the human microbiome, cross-sectional, case–control and longitudinal studies may not have enough statistical power to allow causation to be deduced from patterns of association between variables in high-resolution omic datasets. Therefore, to move beyond reliance on the empirical method, experiments are critical. For these, robust experimental models are required that allow the systematic manipulation of variables to test the multitude of hypotheses, which arise from high-throughput molecular studies. Particularly promising in this respect are microfluidics-based *in vitro* co-culture systems, which allow high-throughput first-pass experiments aimed at proving cause-and-effect relationships prior to testing of hypotheses in animal models. This review focuses on widely used *in vivo*, *in vitro, ex vivo* and *in silico* approaches to study host-microbial community interactions. Such systems, either used in isolation or in a combinatory experimental approach, will allow systematic investigations of the impact of microbes on the health and disease of the human host. All the currently available models present pros and cons, which are described and discussed. Moreover, suggestions are made on how to develop future experimental models that not only allow the study of host-microbiota interactions but are also amenable to high-throughput experimentation.

## Review

### Introduction

A human individual’s microbiota consists of around 100 trillion cells, which represent at least ten times as many cells as human cells constitute the body. These microbiota colonize the surface and deep layers of the skin, are found in saliva and the oral cavity, and in the conjunctiva as well as in the gastrointestinal tract (GIT) [1]. Recent large-scale metagenomic sequencing efforts, including those led by the human microbiome project (HMP; National Institutes of Health initiative) [2,3] and the metagenomics of the human intestinal tract (MetaHIT) [4,5] consortia, have convincingly corroborated the notion that humans should be considered as superorganisms in which the microbial symbionts play essential physiological functions [6,7]. Beneficial effects of the presence of microbial communities on human physiology range from immune cell development and homeostasis [8-10], food digestion *via* the fermentation of non-digestible dietary components in the large intestine [11-14] to balancing the host’s metabolism [15-17] and promoting angiogenesis [18,19]. Negative consequences for the host linked to the microbiota include for example chronic inflammation and infection (for recent reviews see [20,21]). Furthermore, shifts in microbial community structure and function (dysbiosis) have been linked to numerous human diseases, including inflammatory bowel disease, diabetes mellitus, obesity, cardiovascular disease and cancer (recently reviewed in [22]).

The largest microbial reservoir of the human body is the GIT and, thus, it is also the most studied and important from a biomedical perspective [23]. Extensive analyses of small subunit (16S) ribosomal RNA gene sequences amplified from fecal samples [24-28], mainly reflecting the luminal microbiota of the distal large intestine, have more recently been supplemented by comprehensive data from large-scale metagenomic sequencing studies to establish a catalogue of microbial organismal and functional diversity in the GIT [4,5,7]. Descriptions at a more detailed taxonomic level reveal many hundreds of species and even more strains in a typical fecal sample [29]. Even though substantial inter-individual variation in microbial community composition has been reported [30], common sets of microbial species include members of the genera *Faecalibacterium*, *Ruminococcus*, *Eubacterium*, *Dorea*, *Bacteroides*, *Alistipes* and *Bifidobacterium*[4,25].

Beyond metagenomics, functional omic approaches, such as metatranscriptomics, metaproteomics and metabolomics (jointly referred to here as ‘meta-omic’ approaches), are now also rapidly gaining pace [31-33]. Integrated omic approaches provide qualitative and quantitative information on genetic potential, transcripts, proteins and metabolites that are present in microbial communities at specific points in space and time [34]. Moreover, such approaches have the potential to highlight the systemic impact of microbial communities beyond the vicinity of the GIT and thereby highlight the intricate cross-feeding between both the human and microbial (eco-)systems. For example, Wikoff and coworkers showed using a metabolomic approach that amino acid metabolites found in mammalian blood were particularly affected by the GIT microbiome [35]. In particular, indole-3-propionic acid (derived from tryptophan) could only be detected in conventionally raised mice compared to germ-free mice [35]. Only after colonization of the germ-free mice with *Clostridium sporogenes* could this bioactive amino acid derivate be identified in the plasma of these animals [35]. Another example of metabolic cross-feeding evidenced by metabolomics includes the higher prevalence of tricarboxylic acid cycle (TCA) metabolites in conventionally raised mice *versus* germ-free mice [36].

**Figure 1:**
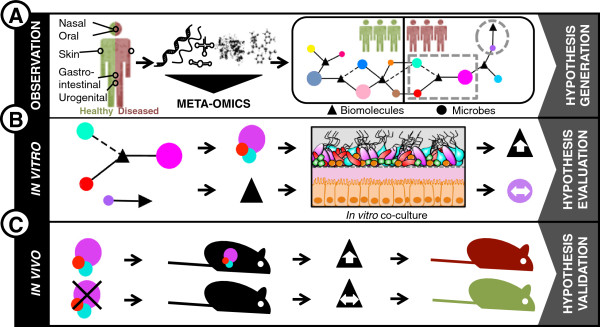
**From association to causality.** (**A**) Functional co-occurrence networks, established by the analysis of human microbial communities from healthy and diseased cohorts by meta-omic approaches are crucial to define dysbiotic states and to correlate individual microbial community members with disease. (**B**) In order to gain detailed information about microbial compositional changes and their associated impact on disease, high-throughput *in vitro* experimental systems are essential. *In vitro* co-culture approaches allow the confirmation or the rejection of hypotheses resulting from meta-omic data. (**C**) In order to causally link changes in microbial community structure or in their associated biomolecular patterns with specific diseases, gnotobiotic animal models are indispensable for *in vivo* validation. In all panels, triangles represent different biomolecules whereas color-coded circles represent different microbial taxa.

It is clear that ‘meta-omics’ approaches result in data that is essential for the definition of baseline healthy microbiota and the identification of differences that may be associated with human disease [37]. Recently, integration of different ‘meta-omic’ approaches was successfully used to pinpoint bacterial members of the GIT community, which are active, damaged, or responsive to a given compound [38]. However, to causally link the identified differences in the human microbiota with distinct human phenotypes including diseases, experiments are essential (Figure 1A). Therefore, representative *in vitro* (Figure 1B) and *in vivo* (Figure 1C) experimental models are required, which allow the systematic manipulation of variables and, thus, allow experimental testing and validation of results derived from meta-omics.

### *In vivo* models

Germ-free (GF) animals are reared in sterile isolators to control their exposure to microorganisms, including viruses, bacteria and eukaryotic parasites [39]. If these animals are colonized with specific microorganisms they become gnotobiotic [39]. The colonization of GF animals mimics the birth of an infant from a sterile environment of the womb towards a microbe-dominated environment. A considerable advantage of GF animals is that they can be made gnotobiotic with GIT microbial communities of specific donors (human or other animal species) and therefore allow analysis of the systemic impact of specific microorganisms on the xenograft recipient. Indeed, colonization of GF animals with single individual bacterial species allows one to directly link a putative function or shaping of the GIT to a particular bacterial species or group [40,41]. Being able to associate a specific function to a particular bacterial strain or species is of utmost importance considering the possibility that specific organisms could be used as a treatment for a given disease. However, a major drawback of monocolonization of GF animals with specifically selected bacteria is the lack of scope of such investigations to uncover the full effects of single bacterial species on the host because of lack of full microbial community context in such approaches. Therefore, the beneficial impacts of a single bacterial species/strain on the host should be validated by taking into account the full microbial community context. In particular, shifts in microbial community structure need to be assessed when adding single strains to communities to avoid potentially triggering a dysbiotic state.

Different GF animal models (zebrafish, mice, rats and even pigs) have already been successfully established (Tables 1 and 2). Undoubtedly, such animal models have proven to be essential for studying host-microbe interactions (in particular immune-microbe feedbacks) and have allowed causal links to be established in a limited number of studies [42,43].

**Table 1:** Advantages and disadvantages of different animal models commonly used for studying host-microbe interactions

**Animal model**	**Advantages**	**Disadvantages**
**Zebrafish**	• Transparent until adulthood allowing real-time visualization of fluorescently labeled microbes throughout the gut [44].	• GIT is homologous to that of mammals but not identical (reviewed in [45]).
	• Chemical screens and forward genetic tests can be performed to investigate host genetic factors or signaling pathways regulated by microbes [46].	• Diet and living environment strongly differs from humans
	• Relatively short generation time (3 to 4 months) with a progeny size of about 100 to 200 eggs/female [46].	• Aging differs strongly from humans
	• 3 to 4 cm long as an adult allowing storage of a large number in laboratory facilities [46].	• Zebrafish and their natural pathogens exist at a temperature of 28°C, while most human-relevant pathogens are only infectious at 37°C [47].
	• Genome fully sequenced (http://www.sanger.ac.uk/Projects/D_rerio/).	• Zebrafish do not have distinguishable lymph nodes, Peyer’s patches, or splenic germinal centers [48].
	• Well characterized mutant strains [46].	
	• Gastrointestinal tract (GIT) is homologous to that of mammals, containing a liver, pancreas, gall bladder, and a linearly segmented intestinal tract with absorptive and secretory functions. The intestinal epithelium forms tight junctions and microvilli. Displays absorptive enterocytes, goblet cells, and enteroendocrine cells (reviewed in [45]).	
**Mouse**	• Numerous mouse specific disease models or genetically altered mice are available [49].	• Marked differences in the immune system [50]
	• Well characterized model; genome fully sequenced (http://www.sanger.ac.uk/resources/mouse/genomes/) and virtually all mouse genes have human homologues.	• Marked differences in microbiota composition between mice and humans have been noted [51].
	• Relatively small and thus can be easily maintained.	• Diet and living environment differs from human.
	• Reproduction rather quick so that several generations can be observed in a relatively short period of time, generally a mouse can live 2 to 3 years.	
	• Mice present the same organs as humans but in different proportions	
**Rat**	• A lot of rat-specific disease models or genetically altered rats are available (http://rgd.mcw.edu/wg/physiology).	• Diet and living environment differs from human.
	• Genome fully sequenced (http://rgd.mcw.edu/).	
	• Relatively small and thus can be easily maintained.	
	• Reproduction rather quick so that several generations can be observed in a relatively short period of time,	
	• Generally a rat can live 2 to 3 years.	
**Pig**	• Omnivore.	• Reproduction rather slow (4 months gestation), generally a pig can live 10 to 15 years.
	• Physiology of digestion, digestate transit times and associated metabolic processes are very similar between humans and pigs (reviewed in [52]).	• Important in size and thus expensive and complicated to maintain in laboratory conditions.
	• Digestive tract shares many anatomical and physiological traits with that of humans.	
	• Immune system similar to humans.	
	• Genome fully sequenced (http://www.sanger.ac.uk/resources/downloads/othervertebrates/pig.html).	
	• Conserved homology between human and pig genomes.	

**Table 2:** Examples of successfully conducted microbiota transplantation experiments into germ-free (GF) recipient animals

**Animal model**	**Host microbiota**	^**a**^**GF status *versus *** **CONV-R**^**b **^**animals**	**Donor’s microbiota in the recipient animal**
**Zebrafish**	Predominantly *Proteobacteria* (82% ± 22%) and *Fusobacteria* (11% ± 15.2%). Minor populations are: *Firmicutes*, *Bacteroidetes*, *Verrucomicrobia*, *Actinobacteria*, candidate phyla TM6 and TM7, Planctomycetes, *Nitrospora* and candidate division OP10 [53,54].	Reduced rates of epithelial proliferation [53].	**Mouse to zebrafish**: predominantly *Firmicutes* and *Bacteroides*[51]; when GF zebrafish are colonized with mouse microbiota the transplanted community resembles its community of origin in terms of the lineages present, but the relative abundance of the lineages changes to resemble the normal gut microbial community composition of the recipient host. Thus, a selective pressure of the host is imposed to the recipient’s gut habitat [54].
		Compromised ability to use nutrients [53].	
**Mouse**	Mouse and human microbiota are similar at the phylum level, but different at the genus level (>99% of the bacterial phylogenetic types from wild-type mice belong to two divisions: *Firmicutes* and *Bacteroidetes*) [51].	Most widely used GF animal model and theoretically any mouse strain can be derived to GF status.	**Human to mouse**: Transplantation of fresh or frozen adult human fecal microbial communities into GF mice results in stably and heritably colonized mice with a microbiota that reproduces much of the bacterial diversity of the donor’s microbiota (all bacterial phyla, 11/12 bacterial classes, and 88% (58/66) of genus-level taxa) [55,56].
		Numerous immunological differences in GF animals: Peyer’s patches are poorly formed; composition of CD4^+^ T cells and IgA-producing B cells in the lamina propria is altered [57-63]; impaired development of follicular B- and T-cell areas of the spleen and peripheral lymph nodes [60]. Th17 and Treg CD4^+^ T cells are less efficient in GF mice [61-63].	
		The epithelial cell turnover is decreased by a factor 2 in GF animals compared to CONV-R mice [64,65].	**Obese mouse to GF mouse**: Microbiota transplantation experiments utilizing genetically obese *ob/ob* mice [66],CONV-R mice fed a Western diet [67], and mice lacking the Toll like receptor 5 [68] have demonstrated that colonization with an obesity-associated gut microbiota results in an increased gain in adiposity relative to colonization with a gut microbiota harvested from lean controls.
		Postnatal gene expression of β1-4-galactosyltransferase stays at low levels in GF mice [69] and in general host gene expression differs between GF and colonized mice [70].	
		Compromised ability to use nutrients in GF animals compared to CONV-R mice [60].	**Pig to mouse**: when GF mice are colonized with pig microbiota the overall bacterial group distributions are similar, but colony and cell morphologies of bacteria grown on specific media are different between pig and gnotobiotic mice [71].
		Difference in metabolic signatures in GF mice compared to CONV-R mice and humans [72,73].	
**Rat**	Rat and human microbiota are similar at the phylum level but different at the genus level (wild-type rat microbial communities harbor at least eight different divisions dominated by two major phyla: *Firmicutes* (74%) and *Bacteroidetes* (23%)) [74].	Difference in metabolic signatures [75-81].	**Human to rat**: when GF rats are colonized with human microbiota the transplanted community resembles its community of origin in terms of the group or genus levels but differences at the dominant species level occur. Thus, a selective pressure of the host is imposed on the gut habitat [82]. However, certain metabolic characteristics (high equol-producing and low equol-producing status or cholesterol-to-coprostanol conversion) of human intestinal floras can be transferred to GF rats [82,83].
		In CONV rats, the colonic mucus layer is twice as thick as in GF rats [84], and the mucin chemical composition is altered [85,86].	
		Numerous immunological defects; the proportion of intraepithelial CD4+ and CD8+ T cells is altered [59,87], as well as the composition and T cell receptor repertoire [59].	
		Decreased enterocyte production [88].	
**Pig**	The microbiome of pigs is dominated by two major phyla: *Firmicutes* (≈81%) and *Bacteroidetes* (≈11%) [89,90].	Host gene expression differs between GF and colonized pigs [91].	**Human to pig**: pigs seem to induce less host specific selection of the donor microbiota [92].
		Epithelial cell proliferation and differentiation genes are downregulated in GF piglets compared to CONV-R pigs [91].	**CONV-R pigs to GF pigs**: Genes involved in biological processes such as epithelial cell turnover, nutrient transport and metabolism, xenobiotic metabolism, JAK-STAT signaling pathway, and immune responsiveness become upregulated by the colonization of GF pigs [91].

^a^GF, germ-free.

^b^Conv-R, conventionally raised.

From the most fundamental point of view, the study of GF animals demonstrated that life without microbes is possible [39]. Nevertheless, GF animals show a number of important physiological differences when compared to conventionally raised (CONV-R) animals (Table 2). For example, GF animals show a slower epithelial renewal [64,93], an altered immune system [57-63,94] and marked differences in gene expression of mammalian gastrointestinal cells [69,70,91]. Interestingly, GF animals require a higher caloric intake to maintain their body weight, which might be linked to less efficient whole-body metabolism compared to CONV-R animals [6,72,73,75-81,84,95]. Moreover, GF animals, at least GF rats, exhibit a decreased mucus layer in the GIT compared to CONV-R animals, which might be due to the fact that different bacterial strains stimulate the secretion of colonic mucin [85,86,96]. Thus, since a number of essential physiological functions significantly differ between GF and CONV-R animal models, data extrapolated from experiments carried out with GF animal models must be considered with caution (Tables 1 and 2). However, the comparison between an ‘all’ (CONV-R animals) or ‘nothing’ (GF animals) situation is still invaluable for the definition of the host’s physiological pathways and functions, which are impacted and/or influenced by different microbiota (reviewed in [60]), as well as for the characterization of the biogeography of the GIT [97].

Although different animal models have been developed (Table 1), mice represent the most widely used and best characterized model organisms. Therefore, we chose to illustrate the pros and cons of experimental models by highlighting investigations carried out in GF mice. Several research groups have, for example, investigated how monocolonization by individual bacterial species shape the immune system of the host. In such studies, investigators have demonstrated that colonization of GF mice with particular segmented filamentous bacteria (SFBs) is sufficient to induce Th17 cell populations in the lamina propria of the gut [62,63]. This specific T cell subpopulation, which is important for protecting the host from bacterial and fungal infections, is absent or marginally detectable in GF mice [62,63]. Interestingly, it was recently shown that human colonization with SFBs, in contrast to mice, seems to be age-dependent with the majority of individuals loosing SFBs by the age of 3 years [98]. Moreover, colonization of GF mice with human gut microbiota supplemented with SFB only results in a minor increase in the number of intestinal T cells. This indicates that multiple microbial species are involved in the development of intestinal T cells in humans [99] and that most likely different species and strains are important in humans compared to mice. Similarly, colonization of GF mice with indigenous *Clostridium* spp. [61] or *Bacteroides fragilis*[100] by means of a protease-resistant capsular polysaccharide, can increase the frequency or function of colonic CD4^+^ T regulatory cells, which in turn play critical roles in the maintenance of immune homeostasis [8]. However, these findings have yet to be fully validated in humans.

A particularly interesting animal model for investigating microbes-host immune system feedbacks is the reversible microbe colonized GF mouse model that was recently described by Hapfelmeier *et al.*[101]. The reason behind the development of this model is that the continuous presence of commensal intestinal bacteria has made it difficult to study mucosal immune dynamics (for example, kinetics and longevity of IgA’s), which in turn does not allow answering fundamental questions such as: Is constant bacterial exposure required to induce antibody production? Is repetitive contact of the same bacterial species with the host immune system required to provide specific antibodies? What is the minimal bacterial concentration required to induce antibody production? This model consists of GF mice, colonized with a triple mutant *Escherichia coli* strain, which cannot divide and persist *in vivo* but which can sustain intestinal colonization up to 48 hours. Using this model the authors could decipher the dynamics of IgA immune responses and were able to demonstrate that (i) induction of high-titer IgA can be uncoupled from permanent intestinal bacterial colonization, (ii) an intrinsic threshold exists between 10^8^ and 10^9^ bacteria in the GIT below which the intestinal IgA system shows no response, (iii) the intestinal IgA system lacks classical immune memory characteristics (no observable prime-boost effect), and (iv) the intestinal IgA repertoire represents the most dominant species currently present in the intestine [101].

Using analogous GF approaches, relationships between diet, gut microbial ecology and energy balance have also been investigated (recently reviewed in [21,22,102]). For example, it was demonstrated that transplantation of the cecal microbiota from obese mice fed on high-fat diets into GF recipients increases adiposity, thereby demonstrating a causal relationship between certain microbiota compositions and the host’s energy-harvesting capacity [67].

From GF animals experiments it is now well established that microbial communities impact deeply on essential physiological functions of the host. However, it is oftentimes difficult to causally link an apparent dysbiotic state to disease in humans mainly because of marked differences between mouse and man (see also paragraph below). In a few cases, however, causality within the context of dysbiosis-linked diseases has been demonstrated using microbial transplantation experiments. For example, a colitis phenotype was transferable from *Tbx21*^*−/−*^*/Rag2*^*−/−*^ mice, which develop ulcerative colitis in a microbiota-dependent manner, to wild-type mice by adoptive transfer of the implicated microbiota [42]. Similarly, there is also evidence that an altered microbiota associated with a colitogenic phenotype isolated from NLRP6 inflammasome-deficient mice alone is sufficient to drive intestinal inflammation [43].

While all GF as well as disease-specific CONV-R animal models present a number of advantages for studying host-microbe interactions, they often do not yield reliable preclinical results that readily translate into effective human treatments. Two important factors contribute to this failure: i) on the microbial side, bacterial species that colonize the GIT appear to be host-dependent and, thus, a host-specific microbiota is critical for a given host [99]; and ii) on the host side, the immune responses in non-human mammalian species is oftentimes distinct from those seen in human [50,103]. A partial solution to this problem could be the use of animals that show a humanized immune system. These animal models can be generated by grafting immunodeficient animals with suspensions of hematopoietic progenitor cells and/or human peripheral blood cells, and potentially even with supplemental human tissues driving the generation of human immune cells [104-107]. However, in order to causally link dysbiosis with human diseases, these animal models need to be gnotobiotically transplanted further with ‘humanized’ microbiota. The latter poses additional potential pitfalls, as many microbial species have evolved to fill host-specific niches [55,82,97] and the topology of the mouse GIT is, for example, distinct from that of humans, rendering the mimicking of human-specific niches challenging if not impossible. Consequently, xenograft microbiota may not representatively colonize the GIT of a humanized animal model.

Another conceivable approach to study the interplay between host and microbes is the use of CONV-R animal models treated with antibiotics, leading to a temporary knock-out effect of selected bacterial groups, followed by a repopulation of host GIT with human feces-derived microbial communities. The assumption behind this idea is that these animals do not present significant alterations in essential physiological processes described for GF animals and that the antibiotic intake does significantly enhance the reshaping effect of the transplanted human microbiota. Surprisingly, the combination of antibiotic and transplantation treatments does not increase the establishment of the donor phylotypes but does interfere with the establishment of the exogenous communities by a yet unknown mechanism [74].

In summary, animal models, especially humanized GF models, can be attractive tools for human microbiome research. However, in addition to some of the pitfalls discussed above, animal models are labor-intensive (there is an immense logistical challenge associated with keeping the animals GF), relatively expensive, tedious, and limited in high-throughput. The establishment of animal models that are widely applicable realistic models of human diseases and that could be used to study the specific interplay between microbiota and their host presents a formidable challenge. Therefore, *in vitro* human-microbial co-culture strategies might offer alternative and complementary strategies, since they have a unique potential to facilitate much needed high-throughput validation of hypotheses that are emerging from state-of-the art molecular data and that link certain microbial community compositions and functions to human disease.

### *In vitro* models

*In vitro* models mimic microbial processes along the GIT by employing either distinct serially connected bioreactors/microchannels or a single bioreactor/microchannel mimicking specific part(s) of the human GIT (Table 3). Such models represent enticing alternatives over *in vivo* models because they are typically cheaper and offer greatly improved throughput, flexibility and scalability for hypothesis testing. Moreover, downstream high-resolution molecular analyses are more readily carried out on *in vitro* generated samples compared to those derived from *in vivo* experiments. Traditional *in vitro* systems are however usually based around the partitioned cultivation of specific microbiota in dedicated bioreactors connected in series [108]. They typically lack human cells because they have been developed to model the individual steps catalyzed by the microbiota along the human GIT.

**Table 3:** ***In vitro *** **models used to study host-microbes interactions**

**Feature**	**Transwell Inserts**	**SHIME **[109]	**M-SHIME **[110]	**TIM1 **[111]	**TIM2 **[112]	**CCS **[113]	**Gut-on-a-Chip **[44]
**Human cell culture**^**a**^	+^b^	-	-	-	-	+^b^	++^b^
**GIT microbiota culture**	-	+	+	-	+	-	-
**Anaerobic conditions for microbes**	-	+	+	+	+	+	-
**Mucin**	+	-	+	-	-	-	-
**pH measurement**	-	+	+	+	+	+	-
**Throughput**	+	-	-	-	-	-	+

^a^The models involving the possibility to incorporate human cells can be subjected to Trans-epithelial Electric Resistance (TEER) measurements (except the model CCS).

^b^+, co-culture for 3 to 6 h; ++, co-culture for >1 week.

CCS, continuous culture system; GIT, gastrointestinal tract; M-SHIME, Mucus-Simulator of the Human Intestinal Microbial Ecosystem; SHIME, Simulator of the Human Intestinal Microbial Ecosystem; TIM1, model simulating the stomach and small intestine; TIM2, model simulating the large intestine.

A pioneering example of such an *in vitro* system is the Simulator of the Human Intestinal Microbial Ecosystem (SHIME) [109]. Five reactors, harboring mixtures of luminal microbes, are sequentially connected to mimic acid- and pepsin-mediated digestions in the stomach, metabolism of monosaccharides in the small intestine as well as the distinct microbial fermentative processes that occur in the ascending, transverse and descending colons, respectively. Recently, a mucus layer has been integrated in the SHIME model (M-SHIME) that allows improved simulation of the mucosal and luminal microbiota in the GIT [110]. The SHIME and M-SHIME models allow the study of gut microbiota using either specific isolates or mixed fecal inocula from healthy and diseased (for example, Crohn’s disease) donors. Altogether, these models can be used to examine roles of the GIT microbiota in the digestion of specific human food ingredients (for example, fermentation of arabinogalactan, xylan and pectin [114]), to understand the pharmacokinetics of drugs (for example, sulphasalazine [114]) and/or to model the gut microbiota linked to gastrointestinal disorders [115].

Another well-established model is the *in vitro* GI tract system (TIM1 and TIM2) [111,112]. These automated models simulate the actions that occur along the GIT with peristaltic mixing as well as the absorption of water and fermentation products [111,112]. The TIM1 model simulates the stomach and the small intestine [111], whereas TIM2 mimics the large intestine [112]. The unique characteristics of these models are their unique capability to reflect a drug’s bioavailability in the intestine. Other unique characteristics include the modeling of the luminal conditions in the GIT of humans and monogastric animals by taking into account the secretion of gastric and small bowel fluids, GI transit times and discharge of microbially metabolized compounds. Therefore, similar to the SHIME model, these models allow investigations of the metabolic capability of the effective microbiota in the GIT. However, the TIM models clearly lack the possibility of partitioning the luminal and mucosal microbiota in artificial niches that reflect the GIT and, thus, cannot be regarded as fully representative models.

A third example of an *in vitro* model is a three-stage continuous culture system (CCS), which was originally designed to study the effect of mucin on microbial sulfate reduction and methanogenesis [113]. The highlight of this model is that it reproduces some of the nutritional features, pH characteristics and fluid retention times of the large intestine with each vessel having a different operating volume and pH. Three different vessels are interconnected and mimic the microbial activities in the cecum, transverse colon and descending colon by taking into account the following characteristics: 1) the cecum is a nutrient-rich environment and has microbial growth at low pH, and 2) the other two subparts of the colon are rather nutrient limited with slow microbial growth at neutral pH. Recently, co-cultures of bacterial communities isolated out of a CCS and human intestinal Caco-2 cells were sustained for approximately 3 h in order to study bacterial adhesion to epithelial cells and to measure bacteria-triggered cytokine release [116,117].

A conceptual colonic fermentation model that is analogous to CCS - three similar sized reactors are connected in a continuous culture system mimicking the proximal, transverse and descending colons each at a distinct pH - has recently been suggested by Payne *et al.*[118]. Human fecal microbiota, either planktonic or immobilized on polysaccharide beads, can be inoculated in the proximal reactor and, thus, the metabolic transformations of different nutrients can be investigated. The results can be compared with the metabolism of the same nutrients in a batch-type reactor mimicking only one compartment and, thus, highlight the relative importance of the different compartments for the digestion of specific nutrients.

Although the above-mentioned *in vitro* models have been successfully used to address specific research questions, they present a number of limitations, which hinder their routine use for the study of versatile questions related to the human GIT microbiome. The major drawback of all the models is the lack of long term co-cultures of human and microbial cells, and the subsequent inability to investigate questions related to host-microbe interactions [102,109]. Furthermore, all of these models are limited in the scope of hypotheses that can be tested. The former shortcoming might be taken care of by using Transwell inserts, in which microbial and human cell cultures can be separated by semipermeable membranes. This arrangement should theoretically allow continuous co-cultures of human cells with microbial consortia from the respective bioreactor compartments. However, the Transwell cell cultures can only be employed as end-point assays [119-122] and bear ample risks for cross-contamination of cultures in human-microbe co-culture experiments. Most importantly, however, the representative inclusion of strictly anaerobic microbiota in Transwell insert setups is not possible.

In contrast, adaptation of microfluidics-based cell culture approaches provide important characteristics for development of improved GIT *in vitro* models. These characteristics include laminar flow profiles, small volumes, continuous diffusion-based perfusion, controlled chemical gradients and the ability to probe cells in spatial confinements mimicking their extracellular matrix *in vivo*[123-125]. Most of these characteristics are unachievable in macro-scale bioreactor setups but they are essential for the ability to directly co-culture human and microbial cells under physiologically relevant conditions [126]. The ability to co-culture human and microbial cells is particularly important to understand the intricate interplay between the human and microbial components that might be driven by direct cell-cell interactions and/or extracellular signaling. Naturally, these interactions have a marked impact on all processes related to the human microbiome and, thus, should be incorporated in any representative *in vitro* model. Consequently, pronounced interest currently exists for the development of microfluidics-based *in vitro* models of the human GIT [127], in particular models that allow human-microbial co-cultures. The enormous potential of such approaches has recently been demonstrated by a study focusing on host-pathogen interactions [128], and by the successful co-cultivation of symbiotic microbial communities in aqueous micro-droplets that were probed for synergistic interactions [129]. Conversely, *in vitro* (micro-)fluidics-based systems have so far been mainly used for studying medically relevant biofilm formation using microbial isolate cultures [130-132]. Although several research groups have co-cultured different human cell types [133,134] only a limited number of studies have reported the successful co-culture of human cells with microbial isolates [135-138]. Microfluidic cell co-culture devices typically incorporate semipermeable membranes or porous materials that allow cell feed to diffuse through the permeable barrier to the cells, thereby protecting them from shear stress while simultaneously allowing exchange of nutrients and waste products. Efforts have recently been made to culture multiple cell types across such permeable barriers thereby allowing the mimicking of tissue niches [139]. Incorporation of protective yet permeable barriers is especially pertinent when co-culturing human cells and microbiota due to the large differences in their respective growth rates and possible bacterial virulence to their human counterparts [128].

Microfluidics-based *in vitro* human-microbial co-culture models may offer interesting characteristics for conducting rapid first-pass experiments aimed at proving cause-and-effect relationships (Figure 1B). Moving beyond the traditional lab-scale bioreactors, microfluidic models should allow the co-culture of human and microbial cells for extended periods of time and allow targeted perturbation experiments to be carried out.

Most recently, a promising microfluidics-based Gut-on-a-Chip model has been presented that indeed allows the direct co-culture of epithelial cells with probiotic strains [44]. The model includes many dynamic physical and functional features of the human GIT essential for transport, absorption, and toxicity studies. Therefore, it can be regarded as an essential research tool for drug testing [44]. However, the model still lacks important features, most notably a simulated mucosal barrier and the provision for culturing strict anaerobic microorganisms that dominate the human gastrointestinal microbiota.

**Figure 2:**
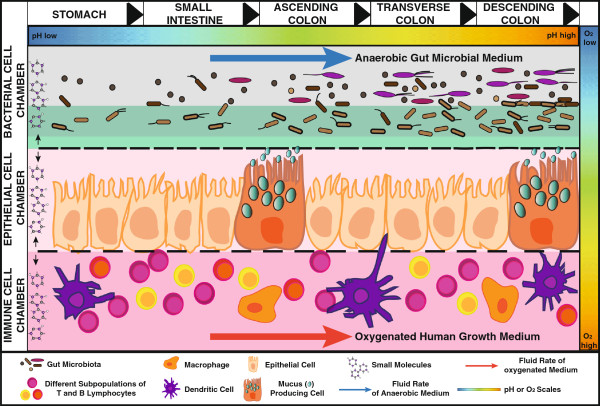
**Conceptualization of an idealized *in vitro *gastrointestinal experimental model.** An idealized *in vitro* co-culture model may include three distinct culture chambers, namely microbial, human epithelial and human immune cell culture chambers, each separated by semipermeable membranes allowing molecular cross-talk between the different contingents while preventing microbes from rapidly overtaking human cells due to pronounced differences in their respective growth rates. Furthermore, an idealized gastrointestinal *in vitro* model should reflect the biogeographical distribution of the gastrointestinal microbiota. Such a model should allow the culture of representative microbial communities for the individual sections of the gastrointestinal tract (GIT) including stomach, small intestine, ascending colon, transverse colon and descending colon. All the individual compartments should be connected in series and allow modulation of their respective environmental factors including pH, fluid retention times, growth medium and other physiological factors such as mucin (in green in the microbial chamber) compositions, which actively interact and alter the microbial communities. To represent the GIT in the most realistic way, the microbial growth chamber needs to be depleted of oxygen, which could be achieved by flushing this chamber with anaerobic microbial medium, whereas the human cell chambers need to be flushed with oxygenated medium. Finally, an idealized GIT *in vitro* model suitable for microbiome research must support high-throughput omic analyses and, thus, needs to allow probing of the individual contingents to perform dedicated analyses on the different cell contingents following a particular experimental regime and to relate particular measurements back to the cell populations of origin.

Nonetheless the Gut-on-a-Chip model has laid the foundation for the development of novel microfluidics-based devices that allow sustained cultivation of human and representative gut microbial communities (also encompassing anaerobes) to study the links between microbial dysbiosis and disease pathogenesis in a truly systematic and representative manner (Figure 2).

### *Ex vivo* models

Besides *in vivo* and *in vitro* experimental models, *ex vivo* models represent interesting tools to study host-microbiota interactions. Such systems compromise explant cultures (tissue cultures) extracted from the colon or rectum [140]. The advantages of such models, is that the tissue cytoarchitecture, including epithelial, lymphocyte subtypes and follicular-dendritic cell networks, is preserved and, thus, such systems can mimic more closely the *in vivo* situation when compared to traditional *in vitro* systems including isolated human cell types. For example, Tsilingiri *et al.* recently described a human mucosa explant culture model in which an apical to basolateral polarity is preserved during stimulation with bacteria [141]. Using this model, the authors demonstrated that probiotics are not always beneficial for the health of the host but that some postbiotics (metabolic products of probiotics) can protect healthy tissue against the inflammatory properties of invasive *Salmonella*[141].

Major drawbacks of *ex vivo* models are that during surgical resection, the mucus layer is at least partially removed or damaged. Additionally, the tissues are generally treated with a cocktail of antibiotics to avoid microbial contamination and therefore no immediate co-culture of microbial communities and human GI cells is achievable.

Furthermore, explants do not offer the required modularity that would allow analyses to be related to specific cell populations but rather only allows links to whole communities of cells, thereby convoluting any relevant molecular signals.

### *In silico* models

To investigate microbe-microbe and host-microbe metabolic interactions, *in silico* reconstructions of genome-scale metabolic networks combined with constraint-based modeling also exhibit promising attributes. The conversion of a metabolic reconstruction (for example, as derived from the human genome sequences, microbial isolate genome sequences and/or metagenomic sequences) into a condition-specific model (for example, GIT model) requires the transformation of a (hopefully) comprehensive biochemical reaction list into a computable, mathematical matrix format [142]. It also requires the addition of physico-chemical constraints (for example, mass conservation) and system boundaries [142]. Thus, (meta-)genome-scale, manually curated metabolic reconstructions can serve as knowledge-bases to summarize existing knowledge about cellular pathways in target organisms in a well-structured, mathematical manner [142]. However, so far, the knowledge-base for microbe-microbe as well as host-microbe interactions is very sparsely populated and extensive dedicated efforts are required in coming years to establish such a database. Consequently, at present, cross-feeding and/or interaction experiments cannot yet be routinely carried out *in silico*.

Even though*, in silico* constructed models represent powerful tools for modeling and predicting phenotypic characteristics of single organisms living in a community within a particular host [143], they can only be constructed based on existing experimental data. Consequently, *in vitro*, *ex vivo* and *in vivo* experimental data, which allow the unraveling of links between gut microbiota and host metabolism, are crucial for constructing comprehensive host-microbe interaction models as well as for the benchmarking of such models. Once constructed, models can predict what role individual microorganisms have and how their actions influence others within their native community [144]. The resulting hypotheses may drive experiments, which will in turn inform and improve the models. Thus, a combined computational-experimental approach for hypothesis generation and testing has the promise to accelerate new discoveries in the realm of microbe-microbe and microbe-host metabolic interactions. Disadvantages of *in silico* models are that the reconstruction process of such genome-scale high quality metabolic networks requires extensive computational and manual analysis efforts and that any new findings need to be experimentally validated. Furthermore, the resulting findings, especially in the case of *in silico* animal-based models [145], may not always efficiently translate to humans [143,145]. Despite the limitations of current *in silico* reconstructed host-microbe interaction models, such approaches are of utmost importance because they theoretically allow a detailed molecular resolution of the complex relationships within microbial communities and with their host. Therefore, such system approaches could dramatically improve our understanding of individual bacterial taxa within communities and the modes of interactions in which they engage. New links between a host and its microbiota could thus be predicted and perturbation experiments (for example, diet variation for the host or changed medium composition for the microbiota) and their outcome could easily be simulated. An example of a successful use of such an *in silico* reconstructed model was recently published by Heinken and co-workers [145]. In this study, the authors reconstructed and analyzed the first integrated stoichiometric model of murine and *Bacteroides thetaiotaomicron* metabolism and could demonstrate *in silico* that even at a low growth rate of *Bacteroides thetaiotaomicron* the mouse profited significantly from the presence of this microorganism in the gut lumen [145].

In summary, host-microbe *in silico* models, when combined with experimental data, will greatly strengthen our knowledge on how microbes influence their host and *vice versa.* Consequently, *in silico* models in combination with *in vitro*, *ex vivo* and *in vivo* experimental data will become an invaluable tool to predict metabolic interactions between gut microbes and their host in both diseased and healthy states.

### Conceptualization of experimental models

A conceptualized ideal experimental model (Figure 2) for the study of host-microbiota interactions in the GIT and one that would allow testing the myriad of hypotheses linking dysbiosis to disease should allow paired wet- and dry-lab experiments and mimic as closely as possible the GIT. Such a model should in particular include: i) human GIT cells; ii) human microbiota sustainably growing under anaerobic/microaerophilic conditions; iii) a mucus layer simulating the physical separation of human and microbial cell contingents; and iv) the physico-chemical conditions encountered in the GIT including primarily pH, fluid retention times and dissolved O_2_ concentrations. Moreover, such a model should reflect simple and controlled experimental settings to allow reproducibility and limit discrepancies in the obtained results due to inter-individual variations occurring in *in vivo* animal models. Finally, in the case of a wet-lab model, it should allow massively parallel screening and validation of results revealed through meta-omic investigations of human subjects.

An *in vitro* living cell-based and microfluidics-based model appears best suited to achieve the highlighted requirements since the gut microenvironment can be simulated by flowing specialized media at defined rates through the respective microchambers seeded with human intestinal cells and microbial communities, respectively. Furthermore, to simulate the physical separation of both human and microbial cells encountered *in vivo*[146], both contingents should be separated either by mucin and/or semipermeable membranes while still guaranteeing molecular cross-talk (metabolites, proteins, nucleic acids, cytokines, chemokines, *etcetera*) between both compartments. The feasibility of such a microfluidics-based *in vitro* model has already been established by the successful co-culture of a single microbial species for over one week on the luminal surface of cultured epithelial cells without compromising epithelial cell viability [44]. However, a future challenge that needs to be overcome in order to be able to reproducibly analyze the interplay between microbes and their host in such an experimental model is the requirement for maintaining aerobic conditions in the human microfluidic chamber while at the same time guaranteeing strictly anaerobic conditions in the microbial chamber, which allow the culture of obligate anaerobic human gut microbes. Finally, an idealized *in vitro* model should include separate compartments mimicking the stomach, small intestine, the ascending colon, the transverse colon and the descending colon, each reflecting the biogeographically distinct characteristics of the GIT.

In summary the conceptualized *in vitro* experimental model could be an elegant supplement to animal *in vivo* and *in silico* models since it would exhibit simple and controlled experimental settings allowing reproducibility and limit discrepancies resulting from inter-individual variations that occur in *in vivo* animal models. Moreover, by the introduction of human cells the interplay between human and microbial cells should be traceable in real-time. This point seems to be of particular importance since a host-specific microbiota appears to be critical for a given healthy host [99]. Finally, the described conceptualized *in vitro* experimental approach would be well suited for high-throughput experiments in contrast to *in vivo* animal models. However, due to their *in vitro* nature experiments carried out in such a conceptualized experimental model will for the most part still require *in vivo* experimental validation. Combined experimental approaches in animal and *in vitro* models could thus lead to the establishment of causal relationships between microbial community compositions and human diseases.

## Conclusions

Understanding of the human gastrointestinal microbiota and its putative role in governing health and disease states has rapidly expanded in recent years. However, the myriads of results, generated by linking ‘meta-omic’ data to disease, still require experimental validation. To reach this goal, we propose a biphasic experimental validation approach: i) rapid first-pass experiments in *in vitro* devices, which allow massively parallel screening and immediate cause-and-effect read-outs (Figure 1B) and ii) *in vivo* experimental validation (Figure 1C).

Microfluidics-based *in vitro* devices appear particularly well suited for high-throughput experiments due to their small footprint, their ability to allow the co-culture of both human and microbial cells for extended periods of time, and their ability to facilitate dynamic perturbation experiments. However, *in vitro* experiments will for the most part still need to be followed up with *in vivo* experiments. Such experimental validation may be achievable using GF animal models. However, owing to specific differences in the living environments of GF and wild-type (CONV-R) animals, the physiologies of both are likely to be distinct, which leads to the somewhat artificial nature of GF animals. Therefore, in order to study the impact of specific microbial strains on the host, gnotobiotic animal models should be directly compared to CONV-R animals. In addition, GF animals should ideally be compared to ex-GF animals colonized by exposing them to the environmental conditions in which CONV-R animals were raised.

We are living through exciting times in human microbiome research. With the advent of high-throughput molecular tools, we are for the first time able to probe the extensive organismal and functional diversity of the human host and identify links between certain microbial community constellations and disease. Beyond this cataloguing effort, experimental validation will become a major component of future studies. Importantly, all the necessary technology for devising representative high-throughput *in vitro* models is available. In the future, such models will become invaluable for large-scale screening efforts prior to *in vivo* experimental and clinical validation.

## Abbreviations

CCS: continuous culture system; CONV-R: conventionally raised; GF: germ-free; GIT: gastrointestinal tract; HMP: Human Microbiome Project; MetaHIT: metagenomics of the human intestinal tract; M-SHIME: Mucus-Simulator of the Human Intestinal Microbial Ecosystem; SFB: segmented filamentous bacteria; SHIME: Simulator of the Human Intestinal Microbial Ecosystem; TCA cycle: tricarboxylic acid cycle; TIM 1: model simulating the stomach and small intestine; TIM 2: model simulating the large intestine.

## Competing interests

The authors declare that they have no competing interests.

## Authors’ contributions

JVF, MSD, PS, JGS, and PW wrote the paper. All authors commented and approved on the final version of the manuscript.

## References

[B1] Luckey TD Introduction to intestinal microecology Am J Clin Nutr 1972 1 1292 1294 4639749 10.1093/ajcn/25.12.1292

[B2] Peterson J Garges S Giovanni M McInnes P Wang L Schloss JA Bonazzi V McEwen JE Wetterstrand KA Deal C Baker CC Di Francesco V Howcroft TK Karp RW Lunsford RD Wellington CR Belachew T Wright M Giblin C David H Mills M Salomon R Mullins C Akolkar B Begg L Davis C Grandison L Humble M Khalsa J Little AR The NIH human microbiome project Genome Res 2009 1 2317 2323 19819907 10.1101/gr.096651.109 PMC2792171

[B3] Markowitz VM Chen I-M A Palaniappan K Chu K Szeto E Grechkin Y Ratner A Anderson I Lykidis A Mavromatis K Ivanova NN Kyrpides NC The integrated microbial genomes system: an expanding comparative analysis resource Nucleic Acids Res 2010 1 D382 D390 10.1093/nar/gkp887 19864254 PMC2808961

[B4] Qin J Li R Raes J Arumugam M Burgdorf KS Manichanh C Nielsen T Pons N Levenez F Yamada T Mende DR Li J Xu J Li S Li D Cao J Wang B Liang H Zheng H Xie Y Tap J Lepage P Bertalan M Batto J-M Hansen T Le Paslier D Linneberg A Nielsen HB Pelletier E Renault P A human gut microbial gene catalogue established by metagenomic sequencing Nature 2010 1 59 65 10.1038/nature08821 20203603 PMC3779803

[B5] Arumugam M Raes J Pelletier E Le Paslier D Yamada T Mende DR Fernandes GR Tap J Bruls T Batto J-M Bertalan M Borruel N Casellas F Fernandez L Gautier L Hansen T Hattori M Hayashi T Kleerebezem M Kurokawa K Leclerc M Levenez F Manichanh C Nielsen HB Nielsen T Pons N Poulain J Qin J Sicheritz-Ponten T Tims S Enterotypes of the human gut microbiome Nature 2011 1 174 180 10.1038/nature09944 21508958 PMC3728647

[B6] Bäckhed F Ding H Wang T Hooper LV Koh GY Nagy A Semenkovich CF Gordon JI The gut microbiota as an environmental factor that regulates fat storage Proc Natl Acad Sci USA 2004 1 15718 15723 10.1073/pnas.0407076101 15505215 PMC524219

[B7] Gill SR Pop M Deboy RT Eckburg PB Turnbaugh PJ Samuel BS Gordon JI Relman D Fraser-Liggett CM Nelson KE Metagenomic analysis of the human distal gut microbiome Science 2006 1 1355 1359 10.1126/science.1124234 16741115 PMC3027896

[B8] Lathrop SK Bloom SM Rao SM Nutsch K Lio C-W Santacruz N Peterson D Stappenbeck TS Hsieh CS Peripheral education of the immune system by colonic commensal microbiota Nature 2011 1 250 254 10.1038/nature10434 21937990 PMC3192908

[B9] Olszak T An D Zeissig S Vera MP Richter J Franke A Glickman JN Siebert R Baron RM Kasper DL Blumberg RS Microbial exposure during early life has persistent effects on natural killer T cell function Science 2012 1 489 493 10.1126/science.1219328 22442383 PMC3437652

[B10] Hooper LV Macpherson AJ Immune adaptations that maintain homeostasis with the intestinal microbiota Nat Rev Immunol 2010 1 159 169 10.1038/nri2710 20182457

[B11] Van Wey AS Cookson AL Roy NC McNabb WC Soboleva TK Shorten PR Bacterial biofilms associated with food particles in the human large bowel Mol Nutr Food Res 2011 1 969 978 10.1002/mnfr.201000589 21638777

[B12] Leitch ECM Walker AW Duncan SH Holtrop G Flint HJ Selective colonization of insoluble substrates by human faecal bacteria Environ Microbiol 2007 1 667 679 10.1111/j.1462-2920.2006.01186.x 17298367

[B13] Ze X Duncan SH Louis P Flint HJ Ruminococcus bromii is a keystone species for the degradation of resistant starch in the human colon ISME J 2012 1 1535 1543 10.1038/ismej.2012.4 22343308 PMC3400402

[B14] Flint HJ Scott KP Louis P Duncan SH The role of the gut microbiota in nutrition and health Nat Rev Gastroenterol Hepatol 2012 1 577 589 10.1038/nrgastro.2012.156 22945443

[B15] Louis P Scott KP Duncan SH Flint HJ Understanding the effects of diet on bacterial metabolism in the large intestine J Appl Microbiol 2007 1 1197 1208 10.1111/j.1365-2672.2007.03322.x 17448155

[B16] Louis P Young P Holtrop G Flint HJ Diversity of human colonic butyrate-producing bacteria revealed by analysis of the butyryl-CoA:acetate CoA-transferase gene Environ Microbiol 2010 1 304 314 10.1111/j.1462-2920.2009.02066.x 19807780

[B17] Louis P Flint HJ Diversity, metabolism and microbial ecology of butyrate-producing bacteria from the human large intestine FEMS Microbiol Lett 2009 1 1 8 10.1111/j.1574-6968.2009.01514.x 19222573

[B18] Stappenbeck TS Hooper LV Gordon JI Developmental regulation of intestinal angiogenesis by indigenous microbes via Paneth cells Proc Natl Acad Sci USA 2002 1 15451 15455 10.1073/pnas.202604299 12432102 PMC137737

[B19] Reinhardt C Bergentall M Greiner TU Schaffner F Ostergren-Lundén G Petersen LC Ruf W Bäckhed F Tissue factor and PAR1 promote microbiota-induced intestinal vascular remodelling Nature 2012 1 627 631 10.1038/nature10893 22407318 PMC3885420

[B20] Turner ML Healey GD Sheldon IM Immunity and inflammation in the uterus Reprod Domest Anim 2012 1 Suppl 4 402 409 22827398 10.1111/j.1439-0531.2012.02104.x

[B21] Nakamura YK Omaye ST Metabolic diseases and pro- and prebiotics: mechanistic insights Nutr Metab 2012 1 60 10.1186/1743-7075-9-60 PMC3464869 22713169

[B22] Pflughoeft KJ Versalovic J Human microbiome in health and disease Annual Review of Pathology 2012 1 99 122 10.1146/annurev-pathol-011811-132421 21910623

[B23] Savage DC Microbial ecology of the gastrointestinal tract Ann Rev Microbiol 1977 1 107 133 10.1146/annurev.mi.31.100177.000543 334036

[B24] Eckburg PB Bik EM Bernstein CN Purdom E Dethlefsen L Sargent M Gill SR Nelson KE Relman D Diversity of the human intestinal microbial flora Science 2005 1 1635 1638 10.1126/science.1110591 15831718 PMC1395357

[B25] Tap J Mondot S Levenez F Pelletier E Caron C Furet J-P Ugarte E Muñoz-Tamayo R Paslier DLE Nalin R Dore J Leclerc M Towards the human intestinal microbiota phylogenetic core Environ Microbiol 2009 1 2574 2584 10.1111/j.1462-2920.2009.01982.x 19601958

[B26] Walker AW Ince J Duncan SH Webster LM Holtrop G Ze X Brown D Stares MD Scott P Bergerat A Louis P McIntosh F Johnstone AM Lobley GE Parkhill J Flint HJ Dominant and diet-responsive groups of bacteria within the human colonic microbiota ISME J 2011 1 220 230 10.1038/ismej.2010.118 20686513 PMC3105703

[B27] Suau A Bonnet R Sutren M Gibson GR Collins MD Doré J Bonnet GIS Dore J Direct analysis of genes encoding 16S rRNA from complex communities reveals many novel molecular species within the human Gut direct analysis of genes encoding 16S rRNA from complex communities reveals many novel molecular species within the human Gut Appl Environ Microbiol 1999 1 4799 4807 10543789 10.1128/aem.65.11.4799-4807.1999 PMC91647

[B28] Hold GL Pryde SE Russell VJ Furrie E Flint HJ Assessment of microbial diversity in human colonic samples by 16S rDNA sequence analysis FEMS Microbiol Ecol 2002 1 33 39 10.1111/j.1574-6941.2002.tb00904.x 19709182

[B29] Schloissnig S Arumugam M Sunagawa S Mitreva M Tap J Zhu A Waller A Mende DR Kultima JR Martin J Kota K Sunyaev SR Weinstock GM Bork P Genomic variation landscape of the human gut microbiome Nature 2013 1 45 50 23222524 10.1038/nature11711 PMC3536929

[B30] Human Microbiome Project Consortium Structure, function and diversity of the healthy human microbiome Nature 2012 1 207 214 10.1038/nature11234 22699609 PMC3564958

[B31] Jansson J Willing B Lucio M Fekete A Dicksved J Halfvarson J Tysk C Schmitt-Kopplin P Metabolomics reveals metabolic biomarkers of Crohn’s disease PLoS One 2009 1 e6386 10.1371/journal.pone.0006386 19636438 PMC2713417

[B32] Verberkmoes NC Russell AL Shah M Godzik A Rosenquist M Halfvarson J Lefsrud MG Apajalahti J Tysk C Hettich RL Jansson JK Shotgun metaproteomics of the human distal gut microbiota ISME J 2009 1 179 189 10.1038/ismej.2008.108 18971961

[B33] Gosalbes MJ Durbán A Pignatelli M Abellan JJ Jiménez-Hernández N Pérez-Cobas AE Latorre A Moya A Metatranscriptomic approach to analyze the functional human gut microbiota PLoS One 2011 1 e17447 10.1371/journal.pone.0017447 21408168 PMC3050895

[B34] Roume H El Muller E Cordes T Renaut J Hiller K Wilmes P A biomolecular isolation framework for eco-systems biology ISME J 2013 1 110 121 10.1038/ismej.2012.72 22763648 PMC3526178

[B35] Wikoff WR Anfora AT Liu J Schultz PG Lesley S Peters EC Siuzdak G Metabolomics analysis reveals large effects of gut microflora on mammalian blood metabolites Proc Natl Acad Sci USA 2009 1 3698 3703 10.1073/pnas.0812874106 19234110 PMC2656143

[B36] Velagapudi VR Hezaveh R Reigstad CS Gopalacharyulu P Yetukuri L Islam S Felin J Perkins R Borén J Oresic M Bäckhed F The gut microbiota modulates host energy and lipid metabolism in mice J Lipid Res 2010 1 1101 1112 10.1194/jlr.M002774 20040631 PMC2853437

[B37] Costello EK Lauber CL Hamady M Fierer N Gordon JI Knight R Bacterial community variation in human body habitats across space and time Science 2009 1 1694 1697 10.1126/science.1177486 19892944 PMC3602444

[B38] Maurice CF Haiser HJ Turnbaugh PJ Xenobiotics shape the physiology and gene expression of the active human gut microbiome Cell 2013 1 39 50 10.1016/j.cell.2012.10.052 23332745 PMC3552296

[B39] Trexler PC Hon D Orcutt RP Development of Gnotobiotics and contamination control in laboratory animal science introduction: nomenclature 50 Years of Laboratory Animal Science. American association for laboratory animal science 1999 Memphis, TN: American Association for Laboratory Animal Science book chapter sixteen 121–128. http://www.aalas.org/association/about.aspx

[B40] Lundell A-C Björnsson V Ljung A Ceder M Johansen S Lindhagen G Törnhage C-J Adlerberth I Wold AE Rudin A Infant B cell memory differentiation and early gut bacterial colonization J Immunol 2012 1 4315 4322 10.4049/jimmunol.1103223 22490441

[B41] Cilieborg MS Boye M Sangild PT Bacterial colonization and gut development in preterm neonates Early Hum Dev 2012 1 Suppl 1 S41 S49 22284985 10.1016/j.earlhumdev.2011.12.027

[B42] Garrett WS Lord GM Punit S Lugo-Villarino G Mazmanian SK Ito S Glickman JN Glimcher LH Communicable ulcerative colitis induced by T-bet deficiency in the innate immune system Cell 2007 1 33 45 10.1016/j.cell.2007.08.017 17923086 PMC2169385

[B43] Elinav E Strowig T Kau AL Henao-Mejia J Thaiss C Booth CJ Peaper DR Bertin J Eisenbarth SC Gordon JI Flavell R NLRP6 inflammasome regulates colonic microbial ecology and risk for colitis Cell 2011 1 745 757 10.1016/j.cell.2011.04.022 21565393 PMC3140910

[B44] Kim HJ Huh D Hamilton G Ingber DE Human gut-on-a-chip inhabited by microbial flora that experiences intestinal peristalsis-like motions and flow Lab Chip 2012 1 2165 2174 10.1039/c2lc40074j 22434367

[B45] Goldsmith JR Jobin C Think small: zebrafish as a model system of human pathology J Biomed Biotechnol 2012 1 817341 22701308 10.1155/2012/817341 PMC3371824

[B46] Patton EE Zon LI The art and design of genetic screens: zebrafish Nat Rev Genet 2001 1 956 966 10.1038/35103567 11733748

[B47] Meeker ND Trede NS Immunology and zebrafish: spawning new models of human disease Dev Comp Immunol 2008 1 745 757 10.1016/j.dci.2007.11.011 18222541

[B48] Danilova N Steiner L B cells develop in the zebrafish pancreas Proc Natl Acad Sci USA 2002 1 13711 13716 10.1073/pnas.212515999 12370418 PMC129751

[B49] Ma B Da L Na J Copeland NG Mouse models of human disease. Part II: recent progress and future directions Genes Dev 1997 1 11 43 10.1101/gad.11.1.11 9000048

[B50] Mestas J Hughes CCW Of mice and not men: differences between mouse and human immunology J Immunol 2004 1 2731 2738 14978070 10.4049/jimmunol.172.5.2731

[B51] Ley RE Bäckhed F Turnbaugh P Lozupone C Knight RD Gordon JI Obesity alters gut microbial ecology Proc Natl Acad Sci USA 2005 1 11070 11075 10.1073/pnas.0504978102 16033867 PMC1176910

[B52] Patterson JK Lei XG Miller DD The pig as an experimental model for elucidating the mechanisms governing dietary influence on mineral absorption Exp Biol Med (Maywood) 2008 1 651 664 10.3181/0709-MR-262 18408137

[B53] Rawls JF Samuel BS Gordon JI Gnotobiotic zebrafish reveal evolutionarily conserved responses to the gut microbiota Proc Natl Acad Sci USA 2004 1 4596 4601 10.1073/pnas.0400706101 15070763 PMC384792

[B54] Rawls JF Mahowald M Ley RE Gordon JI Reciprocal gut microbiota transplants from zebrafish and mice to germ-free recipients reveal host habitat selection Cell 2006 1 423 433 10.1016/j.cell.2006.08.043 17055441 PMC4839475

[B55] Kibe R Sakamoto M Yokota H Aiba Y Koga Y Benno Y Ishikawa H Movement and fixation of intestinal microbiota after administration of human feces to germfree mice movement and fixation of intestinal microbiota after administration of human feces to germfree mice Appl Environ Microbiol 2005 1 3171 3178 10.1128/AEM.71.6.3171-3178.2005 15933018 PMC1151853

[B56] Turnbaugh PJ Ridaura VK Faith JJ Rey FE Gordon JI The effect of diet on the human gut microbiome: a metagenomic analysis in humanized gnotobiotic mice Sci Transl Med 2009 1 6ra14 10.1126/scitranslmed.3000322 20368178 PMC2894525

[B57] Macpherson AJ Harris NL Interactions between commensal intestinal bacteria and the immune system Nat Rev Immunol 2004 1 478 485 10.1038/nri1373 15173836

[B58] Umesaki Y Setoyama H Matsumoto S Okada Y Expansion of alpha beta T-cell receptor-bearing intestinal intraepithelial lymphocytes after microbial colonization in germ-free mice and its independence from thymus Immunology 1993 1 32 37 8509140 PMC1422052

[B59] Helgeland L Vaage JT Rolstad B Midtvedt T Brandtzaeg P Microbial colonization influences composition and T-cell receptor V beta repertoire of intraepithelial lymphocytes in rat intestine Immunology 1996 1 494 501 10.1046/j.1365-2567.1996.d01-783.x 9014812 PMC1456593

[B60] Smith K McCoy KD Macpherson AJ Use of axenic animals in studying the adaptation of mammals to their commensal intestinal microbiota Semin Immunol 2007 1 59 69 10.1016/j.smim.2006.10.002 17118672

[B61] Atarashi K Tanoue T Shima T Imaoka A Kuwahara T Momose Y Cheng G Yamasaki S Saito T Ohba Y Taniguchi T Takeda K Hori S Ivanov II Umesaki Y Itoh K Honda K Induction of colonic regulatory T cells by indigenous Clostridium species Science 2011 1 337 341 10.1126/science.1198469 21205640 PMC3969237

[B62] Ivanov II Atarashi K Manel N Brodie EL Shima T Karaoz U Wei D Goldfarb KC Santee C Lynch SV Tanoue T Imaoka A Itoh K Takeda K Umesaki Y Honda K Littman DR Induction of intestinal Th17 cells by segmented filamentous bacteria Cell 2009 1 485 498 10.1016/j.cell.2009.09.033 19836068 PMC2796826

[B63] Gaboriau-Routhiau V Rakotobe S Lécuyer E Mulder I Lan A Bridonneau C Rochet V Pisi A De Paepe M Brandi G Eberl G Snel J Kelly D Cerf-Bensussan N The key role of segmented filamentous bacteria in the coordinated maturation of gut helper T cell responses Immunity 2009 1 677 689 10.1016/j.immuni.2009.08.020 19833089

[B64] Savage DC Siegel JE Snellen JE Whitt DD Transit time of epithelial cells in the small intestines of germfree mice and transit time of epithelial cells in the small intestines of germfree mice and ex-germfree mice associated with indigenous microorganisms Appl Environ Microbiol 1981 1 996 1001 7198427 10.1128/aem.42.6.996-1001.1981 PMC244145

[B65] Khoury KA Floch MH Hersh T Small intestinal mucosal cell proliferation and bacterial flora in the conventionalization of the germfree mouse J Exp Med 1969 1 659 670 10.1084/jem.130.3.659 4896909 PMC2138714

[B66] Turnbaugh PJ Ley RE Mahowald MA Magrini V Mardis ER Gordon JI An obesity-associated gut microbiome with increased capacity for energy harvest Nature 2006 1 1027 1031 10.1038/nature05414 17183312

[B67] Turnbaugh PJ Bäckhed F Fulton L Gordon JI Diet-induced obesity is linked to marked but reversible alterations in the mouse distal gut microbiome Cell Host Microbe 2008 1 213 223 10.1016/j.chom.2008.02.015 18407065 PMC3687783

[B68] Vijay-Kumar M Aitken JD Carvalho F Cullender TC Mwangi S Srinivasan S Sitaraman SV Knight R Ley RE Gewirtz AT Metabolic syndrome and altered gut microbiota in mice lacking Toll-like receptor 5 Science 2010 1 228 231 10.1126/science.1179721 20203013 PMC4714868

[B69] Nanthakumar NN Dai D Meng D Chaudry N Newburg DS Walker WA Regulation of intestinal ontogeny: effect of glucocorticoids and luminal microbes on galactosyltransferase and trehalase induction in mice Glycobiology 2005 1 221 232 15483270 10.1093/glycob/cwi004

[B70] Hooper LV Wong MH Thelin A Hansson L Falk PG Gordon JI Molecular analysis of commensal host-microbial relationships in the intestine Science 2001 1 881 884 10.1126/science.291.5505.881 11157169

[B71] Hirayama K Itoht K Takahashit E Shinozaki K Sawasaki T Composition of faecal microbiota and metabolism of faecal bacteria of pig-flora-associated ( PFA ) mice Microb Ecol Health Dis 1996 1 199 206 10.3109/08910609609166460

[B72] Claus SP Tsang TM Wang Y Cloarec O Skordi E Martin F-P Rezzi S Ross A Kochhar S Holmes E Nicholson JK Systemic multicompartmental effects of the gut microbiome on mouse metabolic phenotypes Mol Syst Biol 2008 1 219 18854818 10.1038/msb.2008.56 PMC2583082

[B73] Welling GW Groen G Tuinte JH Koopman JP Kennis HM Biochemical effects on germ-free mice of association with several strains of anaerobic bacteria J Gen Microbiol 1980 1 57 63 7391821 10.1099/00221287-117-1-57

[B74] Manichanh C Reeder J Gibert P Varela E Llopis M Antolin M Guigo R Knight R Guarner F Reshaping the gut microbiome with bacterial transplantation and antibiotic intake Genome Res 2010 1 1411 1419 10.1101/gr.107987.110 20736229 PMC2945190

[B75] Meinl W Sczesny S Brigelius-Flohé R Blaut M Glatt H Impact of gut microbiota on intestinal and hepatic levels of phase 2 xenobiotic-metabolizing enzymes in the rat Drug Metab Dispo 2009 1 1179 1186 10.1124/dmd.108.025916 19282396

[B76] Swann JR Want EJ Geier FM Spagou K Wilson ID Sidaway JE Nicholson JK Holmes E Systemic gut microbial modulation of bile acid metabolism in host tissue compartments Proc Natl Acad Sci USA 2011 1 Suppl 4523 4530 10.1073/pnas.1006734107 20837534 PMC3063584

[B77] Nicholls AW Mortishire-smith RJ Nicholson JK Urinary metabolite variation in acclimatizing germ-free rats Chem Res Toxicol 2003 1 1395 1404 10.1021/tx0340293 14615964

[B78] Chin SF Storkson JM Liu W Albright KJ Pariza MW Conjugated linoleic acid (9,11- and 10,12-octadecadienoic acid) is produced in conventional but not germ-free rats fed linoleic acid J Nutr 1994 1 694 701 8169661 10.1093/jn/124.5.694

[B79] Morton KC Wang CY Enhanced macromolecular binding of N-[4-(5-nitro-2-furyl)-2-thiazolyl]- formamide in germfree versus conventional rats Cancer Res 1983 1 3628 3632 6190554

[B80] Ducluzeau R Raibaud P Dickinson AB Sacquet E Mocquot G Hydrolysis of urea in vitro and in vivo, in the cecum of gnotobiotic rats, by different bacterial strains isolated from the digestive tract of conventional rats C R Acad Sci Hebd Seances Acad Sci D 1966 1 944 947 4956274

[B81] Wostmann BS Bruckner-Kardoss E Oxidation-reduction potentials in cecal contents of germfree and conventional rats Proc Soc Exp Biol Med 1966 1 1111 1114 5937711 10.3181/00379727-121-30979

[B82] Gérard P Béguet F Lepercq P Rigottier-Gois L Rochet V Andrieux C Juste C Gnotobiotic rats harboring human intestinal microbiota as a model for studying cholesterol-to-coprostanol conversion FEMS Microbiol Ecol 2004 1 337 343 10.1016/S0168-6496(03)00285-X 19712322

[B83] Bowey E Adlercreutz H Rowland I Metabolism of isoflavones and lignans by the gut microflora: a study in germ-free and human flora associated rats Food Chem Toxicol 2003 1 631 636 10.1016/S0278-6915(02)00324-1 12659715

[B84] Szentkuti L Riedesel H Enss ML Gaertner K Von Engelhardt W Pre-epithelial mucus layer in the colon of conventional and germ-free rats Histochem J 1990 1 491 497 10.1007/BF01007234 1702088

[B85] Meslin JC Fontaine N Andrieux C Variation of mucin distribution in the rat intestine, caecum and colon: effect of the bacterial flora Comp Biochem Physiol A Mol Integr Physiol 1999 1 235 239 10.1016/S1095-6433(99)00056-2 10501018

[B86] Sharma R Schumacher U Morphometric analysis of intestinal mucins under different dietary conditions and gut flora in rats Dig Dis Sci 1995 1 2532 2539 10.1007/BF02220438 8536508

[B87] Helgeland L Dissen E Dai K-Z Midtvedt T Brandtzaeg P Vaage JT Microbial colonization induces oligoclonal expansions of intraepithelial CD8 T cells in the gut Eur J Immunol 2004 1 3389 3400 10.1002/eji.200425122 15517613

[B88] Banasaz M Norin E Holma R Midtvedt T Increased enterocyte production in gnotobiotic rats mono-associated with Lactobacillus rhamnosus GG Appl Environ Microbiol 2002 1 3031 3034 10.1128/AEM.68.6.3031-3034.2002 12039764 PMC123962

[B89] Leser TD Amenuvor JZ Jensen TK Lindecrona RH Boye M Møller K Culture-independent analysis of gut bacteria: the Pig gastrointestinal tract microbiota revisited culture-independent analysis of gut bacteria: the Pig gastrointestinal tract microbiota revisited Appl Environ Microbiol 2002 1 673 690 10.1128/AEM.68.2.673-690.2002 11823207 PMC126712

[B90] Guo X Xia X Tang R Zhou J Zhao H Wang K Development of a real-time PCR method for Firmicutes and Bacteroidetes in faeces and its application to quantify intestinal population of obese and lean pigs Lett Appl Microbiol 2008 1 367 373 10.1111/j.1472-765X.2008.02408.x 19146523

[B91] Chowdhury SR King DE Willing BP Band MR Beever JE Lane AB Loor JJ Marini JC Rund L Schook LB Van Kessel AG Gaskins HR Transcriptome profiling of the small intestinal epithelium in germfree versus conventional piglets BMC Genomics 2007 1 215 10.1186/1471-2164-8-215 17615075 PMC1949829

[B92] Pang X Hua X Yang Q Ding D Che C Cui L Jia W Bucheli P Zhao L Inter-species transplantation of gut microbiota from human to pigs ISME J 2007 1 156 162 10.1038/ismej.2007.23 18043625

[B93] Bates JM Mittge E Kuhlman J Baden KN Cheesman SE Guillemin K Distinct signals from the microbiota promote different aspects of zebrafish gut differentiation Dev Biol 2006 1 374 386 10.1016/j.ydbio.2006.05.006 16781702

[B94] Treiner E Duban L Bahram S Radosavljevic M Wanner V Tilloy F Affaticati P Gilfillan S Lantz O Selection of evolutionarily conserved mucosal-associated invariant T cells by MR1 Nature 2003 1 164 169 10.1038/nature01433 12634786

[B95] Bäckhed F Manchester JK Semenkovich CF Gordon JI Mechanisms underlying the resistance to diet-induced obesity in germ-free mice Proc Natl Acad Sci USA 2007 1 979 984 10.1073/pnas.0605374104 17210919 PMC1764762

[B96] Caballero-Franco C Keller K De Simone C Chadee K The VSL#3 probiotic formula induces mucin gene expression and secretion in colonic epithelial cells Am J Physiol Gastrointest Liver Physiol 2007 1 G315 G322 16973917 10.1152/ajpgi.00265.2006

[B97] Gootenberg DB Turnbaugh PJ Companion animals symposium: humanized animal models of the microbiome J Anim Sci 2011 1 1531 1537 10.2527/jas.2010-3371 20833767

[B98] Yin Y Wang Y Zhu L Liu W Liao N Jiang M Zhu B Yu HD Xiang C Wang X Comparative analysis of the distribution of segmented filamentous bacteria in humans, mice and chickens ISME J 2013 1 615 621 10.1038/ismej.2012.128 23151642 PMC3578561

[B99] Chung H Pamp SJ Hill J Surana NK Edelman SM Troy EB Reading NC Villablanca EJ Wang S Mora JR Umesaki Y Mathis D Benoist C Relman D Kasper DL Gut immune maturation depends on colonization with a host-specific microbiota Cell 2012 1 1578 1593 10.1016/j.cell.2012.04.037 22726443 PMC3442780

[B100] Round JL Mazmanian SK Inducible Foxp3 + regulatory T-cell development by a commensal bacterium of the intestinal microbiota Proc Natl Acad Sci USA 2010 1 12204 12209 10.1073/pnas.0909122107 20566854 PMC2901479

[B101] Hapfelmeier S Lawson ME Slack E Kirundi JK Stoel M Heikenwalder M Cahenzli J Velykoredko Y Balmer ML Endt K Geuking MB Curtiss R McCoy KD Macpherson AJ Reversible microbial colonization of germ-free mice reveals the dynamics of IgA immune responses Science 2010 1 1705 1709 10.1126/science.1188454 20576892 PMC3923373

[B102] Tremaroli V Bäckhed F Functional interactions between the gut microbiota and host metabolism Nature 2012 1 242 249 10.1038/nature11552 22972297

[B103] Legrand N Ploss A Balling R Becker PD Borsotti C Brezillon N Debarry J De Jong Y Deng H Di Santo JP Eisenbarth S Eynon E Flavell R Guzman C Huntington ND Kremsdorf D Manns MP Manz MG Mention J-J Ott M Rathinam C Rice CM Rongvaux A Stevens S Spits H Strick-Marchand H Takizawa H Van Lent AU Wang C Weijer K Humanized mice for modeling human infectious disease: challenges, progress, and outlook Cell Host Microbe 2009 1 5 9 10.1016/j.chom.2009.06.006 19616761 PMC7038630

[B104] Gimeno R Weijer K Voordouw A Uittenbogaart CH Legrand N Alves NL Wijnands E Blom B Spits H Monitoring the effect of gene silencing by RNA interference in human CD34+ cells injected into newborn RAG2−/− gammac−/− mice: functional inactivation of p53 in developing T cells Blood 2004 1 3886 3893 10.1182/blood-2004-02-0656 15319293

[B105] Ito M Hiramatsu H Kobayashi K Suzue K Kawahata M Hioki K Ueyama Y NOD / SCID / gamma null c mouse: an excellent recipient mouse model for engraftment of human cells Blood 2002 1 3175 3182 10.1182/blood-2001-12-0207 12384415

[B106] Melkus MW Estes JD Padgett-Thomas A Gatlin J Denton PW Othieno F Wege AK Haase AT Garcia JV Humanized mice mount specific adaptive and innate immune responses to EBV and TSST-1 Nat Med 2006 1 1316 1322 10.1038/nm1431 17057712

[B107] Traggiai E Chicha L Mazzucchelli L Bronz L Piffaretti J-C Lanzavecchia A Manz MG Development of a human adaptive immune system in cord blood cell-transplanted mice Science 2004 1 104 107 10.1126/science.1093933 15064419

[B108] Kovatcheva-Datchary P Zoetendal EG Venema K De Vos WM Smidt H Tools for the tract: understanding the functionality of the gastrointestinal tract Therap Adv Gastroenterol 2009 1 9 22 10.1177/1756283X09337646 21180550 PMC3002528

[B109] Molly K Vande Woestyne M Verstraete W Development of a 5-step multi-chamber reactor as a simulation of the human intestinal microbial ecosystem Appl Microbiol Biotechnol 1993 1 254 258 7763732 10.1007/BF00228615

[B110] Van den Abbeele P Roos S Eeckhaut V MacKenzie D Derde M Verstraete W Marzorati M Possemiers S Vanhoecke B Van Immerseel F Van de Wiele T Incorporating a mucosal environment in a dynamic gut model results in a more representative colonization by lactobacilli Microb Biotechnol 2012 1 106 115 10.1111/j.1751-7915.2011.00308.x 21989255 PMC3815277

[B111] Minekus M Marteau P Havenaar R Huis INTVELDJHJ A multicompartmental dynamic computer-controlled model simulating the stomach and small intestine ATLA 1995 1 197 209

[B112] Minekus M Smeets-Peeters M Bernalier A Marol-Bonnin S Havenaar R Marteau P Alric M Fonty G Huis in’t Veld JH A computer-controlled system to simulate conditions of the large intestine with peristaltic mixing, water absorption and absorption of fermentation products Appl Microbiol Biotechnol 1999 1 108 114 10.1007/s002530051622 10645630

[B113] Macfarlane GT Gibson GR Cummings JH Comparison of fermentation reactions in different regions of the human colon J Appl Bacteriol 1992 1 57 64 1541601 10.1111/j.1365-2672.1992.tb04882.x

[B114] Molly K Vande Woestyne M De Smet I Verstraete W Validation of the simulator of the human intestinal microbial ecosystem (SHIME) reactor using microorganism-associated activities Microb Ecol Health Dis 1994 1 191 200 10.3109/08910609409141354

[B115] Vermeiren J Van den Abbeele P Laukens D Vigsnaes LK De Vos M Boon N Van de Wiele T Decreased colonization of fecal Clostridium coccoides/Eubacterium rectale species from ulcerative colitis patients in an in vitro dynamic gut model with mucin environment FEMS Microbiol Ecol 2012 1 685 696 10.1111/j.1574-6941.2011.01252.x 22092917

[B116] Bahrami B Child MW Macfarlane S Macfarlane GT Adherence and cytokine induction in Caco-2 cells by bacterial populations from a three-stage continuous-culture model of the large intestine Appl Environ Microbiol 2011 1 2934 2942 10.1128/AEM.02244-10 21378047 PMC3126424

[B117] Bahrami B Macfarlane S Macfarlane GT Induction of cytokine formation by human intestinal bacteria in gut epithelial cell lines J Appl Microbiol 2011 1 353 363 10.1111/j.1365-2672.2010.04889.x 21070518

[B118] Payne AN Zihler A Chassard C Lacroix C Advances and perspectives in in vitro human gut fermentation modeling Trends Biotechnol 2012 1 17 25 10.1016/j.tibtech.2011.06.011 21764163

[B119] Rose W McGowin CL Spagnuolo RA Eaves-Pyles TD Popov VL Pyles RB Commensal bacteria modulate innate immune responses of vaginal epithelial cell multilayer cultures PLoS One 2012 1 e32728 10.1371/journal.pone.0032728 22412914 PMC3296736

[B120] Fukuda S Toh H Hase K Oshima K Nakanishi Y Yoshimura K Tobe T Clarke JM Topping DL Suzuki T Taylor TD Itoh K Kikuchi J Morita H Hattori M Ohno H Bifidobacteria can protect from enteropathogenic infection through production of acetate Nature 2011 1 543 547 10.1038/nature09646 21270894

[B121] Buess M Nuyten DSA Hastie T Nielsen T Pesich R Brown PO Characterization of heterotypic interaction effects in vitro to deconvolute global gene expression profiles in cancer Genome Biol 2007 1 R191 10.1186/gb-2007-8-9-r191 17868458 PMC2375029

[B122] Bhatia SN Balis UJ Yarmush ML Toner M Probing heterotypic cell interactions: hepatocyte function in microfabricated co-cultures J Biomater Sci Polym Ed 1998 1 1137 1160 10.1163/156856298X00695 9860177

[B123] Shah P Vedarethinam I Kwasny D Andresen L Dimaki M Skov S Svendsen WE Microfluidic bioreactors for culture of non-adherent cells Sensors Actuators B: Chemical 2011 1 1002 1008 10.1016/j.snb.2011.02.021

[B124] Meyvantsson I Beebe DJ Cell culture models in microfluidic systems Annu Rev Anal Chem (Palo Alto Calif) 2008 1 423 449 10.1146/annurev.anchem.1.031207.113042 20636085

[B125] Pasirayi G Auger V M Scott S K S M Rahman P Islam M O’Hare L Ali Z Microfluidic bioreactors for cell culturing: a review Micro Nanosystemse 2011 1 137 160 10.2174/1876402911103020137

[B126] Huh D Hamilton GA Ingber DE From 3D cell culture to organs-on-chips Trends Cell Biol 2011 1 745 754 10.1016/j.tcb.2011.09.005 22033488 PMC4386065

[B127] Turnbaugh PJ Ley RE Hamady M Fraser-Liggett CM Knight R Gordon JI The human microbiome project Nature 2007 1 804 810 10.1038/nature06244 17943116 PMC3709439

[B128] Kim J Hegde M Jayaraman A Co-culture of epithelial cells and bacteria for investigating host-pathogen interactions Lab Chip 2010 1 43 50 10.1039/b911367c 20024049

[B129] Park J Kerner A Burns MA Lin XN Microdroplet-enabled highly parallel co-cultivation of microbial communities PLoS One 2011 1 e17019 10.1371/journal.pone.0017019 21364881 PMC3045426

[B130] McBain AJ Chapter 4: In vitro biofilm models: an overview Adv Appl Microbiol 2009 1 99 132 19729092 10.1016/S0065-2164(09)69004-3

[B131] Coenye T Nelis HJ In vitro and in vivo model systems to study microbial biofilm formation J Microbiol Methods 2010 1 89 105 10.1016/j.mimet.2010.08.018 20816706

[B132] Saleh-Lakha S Trevors JT Perspective: microfluidic applications in microbiology J Microbiol Methods 2010 1 108 111 10.1016/j.mimet.2010.03.022 20363265

[B133] Bhatia SN Balis UJ Yarmush ML Toner M Effect of cell-cell interactions in preservation of cellular phenotype: cocultivation of hepatocytes and nonparenchymal cells FASEB J 1999 1 1883 1900 10544172 10.1096/fasebj.13.14.1883

[B134] Stybayeva G Zhu H Ramanculov E Dandekar S George M Revzin A Micropatterned co-cultures of T-lymphocytes and epithelial cells as a model of mucosal immune system Biochem Biophys Res Commun 2009 1 575 580 10.1016/j.bbrc.2009.01.164 19285003 PMC2659419

[B135] Lindén SK Driessen KM McGuckin MA Improved in vitro model systems for gastrointestinal infection by choice of cell line, pH, microaerobic conditions, and optimization of culture conditions Helicobacter 2007 1 341 353 10.1111/j.1523-5378.2007.00509.x 17669108

[B136] Pellicanò A Leone I Imeneo M Amorosi A Luzza F Co-culture of human gastric endoscopic biopsies with Helicobacter pylori: a simple method for studying early phases of bacteria-host interaction J Microbiol Methods 2008 1 346 349 10.1016/j.mimet.2008.05.025 18588923

[B137] Subbiahdoss G Kuijer R Grijpma DW Van der Mei HC Busscher HJ Microbial biofilm growth vs. tissue integration: “the race for the surface” experimentally studied Acta Biomater 2009 1 1399 1404 10.1016/j.actbio.2008.12.011 19158003

[B138] Saldarriaga Fernández IC Busscher HJ Metzger SW Grainger DW Van der Mei HC Competitive time- and density-dependent adhesion of staphylococci and osteoblasts on crosslinked poly(ethylene glycol)-based polymer coatings in co-culture flow chambers Biomaterials 2011 1 979 984 10.1016/j.biomaterials.2010.10.011 20980049

[B139] Skolimowski M Abeille F Nielsen MW Lopacinska JD Molin S Taboryski R Sternberg C Dufva M Geschke O Emnéus J Microfluidic model of cystic fibrosis bronchi Fifteenth International Conference on Miniaturized Systems for Chemistry and Life Science, Seattle, Washington, USA (2011), Curran Associates, Inc 2012 1 2 118

[B140] Grivel J-C Margolis L Use of human tissue explants to study human infectious agents Nat Protoc 2009 1 256 269 10.1038/nprot.2008.245 19197269 PMC3427853

[B141] Tsilingiri K Barbosa T Penna G Caprioli F Sonzogni A Viale G Rescigno M Probiotic and postbiotic activity in health and disease: comparison on a novel polarised ex-vivo organ culture model Gut 2012 1 1007 1015 10.1136/gutjnl-2011-300971 22301383

[B142] Palsson BO Systems biology: properties of reconstructed networks 2006 Cambridge, UK: Cambridge Univ Press 334

[B143] Thiele I Heinken A Fleming RMT A systems biology approach to studying the role of microbes in human health Curr Opin Biotechnol 2013 1 4 12 10.1016/j.copbio.2012.10.001 23102866

[B144] Zengler K Palsson BO A road map for the development of community systems (CoSy) biology Nat Rev Microbiol 2012 1 366 372 22450377 10.1038/nrmicro2763

[B145] Heinken A Sahoo S Fleming RMT Thiele I Systems-level characterization of a host-microbe metabolic symbiosis in the mammalian gut Gut microbes 2013 1 28 40 10.4161/gmic.22370 23022739 PMC3555882

[B146] Ma MG Lindén SK Sutton P Florin TH Mucin dynamics and enteric pathogens Nat Rev Microbiol 2011 1 265 278 10.1038/nrmicro2538 21407243

